# Long-term survival and BRCA status in male breast cancer: a retrospective single-center analysis

**DOI:** 10.1186/s12885-016-2414-y

**Published:** 2016-07-04

**Authors:** Piera Gargiulo, Matilde Pensabene, Monica Milano, Grazia Arpino, Mario Giuliano, Valeria Forestieri, Caterina Condello, Rossella Lauria, Sabino De Placido

**Affiliations:** Department of Clinical Medicine and Surgery, University of Naples Federico II, Via Pansini 5, 80131 Naples, Italy; Lester and Sue Smith Breast Center at Baylor College of Medicine, Houston, Tx USA

**Keywords:** Male breast cancer, BRCA mutations, Survival

## Abstract

**Background:**

Male breast cancer (MBC) is rare. Given the paucity of randomized trials, treatment is generally extrapolated from female breast cancer guidelines.

**Methods:**

This is a retrospective analysis of all male patients presenting with MBC at the Department of Oncology at University Federico II of Naples between January 1989 and January 2014. We recorded the following data: baseline characteristics (age, height, weight, body mass index, risk factors, family history), tumor characteristics (side affected, stage, histotype, hormonal and HER2 status, and Ki-67 expression), treatment (type of surgery, chemotherapy, endocrine therapy, and/or radiotherapy), BRCA1/2 mutation status (if available), other tumors, and long-term survival.

**Results:**

Forty-seven patients were analyzed. Median age was 62.0 [55.0–72.0]. Among risk factors, obesity and family history of breast cancer were associated with 21 % and 30 % of MBC cases, respectively. The majority of tumors were diagnosed at an early stage: stage I (34.0 %) and stage II (44.7 %). Infiltrating ductal carcinoma was the most frequent histologic subtype (95.8 %). Hormone receptors were generally positive (88.4 % of cases were Estrogen receptor [ER] positive and 81.4 % Progesteron receptor [PgR] positive). Human epidermal growth factor receptor 2 (HER2) was positive in 26.8 % of cases; 7.0 % of MBCs were triple negative. The tumor had high proliferation index (Ki67 ≥ 20 %) in 64.7 %. Surgery was predominantly mastectomy (85.1 %), whereas quadrantectomy was performed in 14.9 % of patients. Adjuvant chemotherapy was administered to 70.7 % of patients, endocrine therapy to 90.2 %, trastuzumab to 16.7 % and radiotherapy to 32.6 %. BRCA status was available for 17 patients: 10 wild-type, 1 BRCA1 carrier, 5 BRCA2 carriers, 1 unknown variant sequence. The overall estimated long-term survival was about 90 % at 5 years, 80 % at 10 years and 70 % at 20 years. Patients carrying a BRCA mutation had a significantly lower survival than patients with wild-type BRCA (*p* = 0.04).

**Conclusions:**

Long-term survival was high in MBC patients referred to our clinical unit. Survival was poorer in BRCA-mutated patients than in patients with wild-type BRCA.

## Background

Male breast cancer (MBC) represents about 1 % of all breast cancers and approximately 0.2 % of all male cancers [1, 2]. Its incidence is estimated at <1 per 100,000 men-years, and it appears to be increasing by 1.1 % yearly [1, 2]. However, given the rarity of this disease, few randomized controlled trials have been conducted, and most of the data about MBC come from retrospective studies [3]. Consequently, treatment of MBC is based on female breast cancer guidelines and trials.

Although MBC shares some features of female breast cancer, it differs significantly in terms of epidemiology and biologic features. The etiology of MBC is unclear although anthropometric and hormonal factors appear to be involved in its development. Clinical disorders, such as Klinefelter’s syndrome, obesity, liver diseases and testicular abnormalities, represent risk factors for MBC. These disorders are associated with an imbalanced estrogen/androgen ratio that result in abnormal estrogen exposure [4]. Other risk factors are race and radiation exposure. Moreover, family history and genetic abnormalities, such as mutations of the BRCA1 and BRCA2 genes, play a relevant role in MBC pathogenesis [5].

About 20 % of patients with MBC have a family history of breast cancer. Subjects with a positive first-degree family history have a 2.0-fold increased risk. The risk of MBC increases to more than 10.0-fold if the number of affected first-degree relatives are two (i.e. mother and sister), thus suggesting that genetic factors plays a relevant role in MBC chance [3, 6]. Accordingly, 2 % of patients with MBC develop a second primary breast cancer and more than 20 % of patients develop tumors at other sites, most frequently prostate, colon or genitourinary cancer [3]. The breast cancer susceptibility genes, BRCA1 and BRCA2, are responsible for a high proportion of cases of hereditable breast cancer. Up to 10 % of all MBCs are caused by inherited germline mutations in either of the two BRCA genes [7, 8], mutations in BRCA2 being more frequently recorded in population-based series [8–10]. The estimated lifetime risk of breast cancer is 1–5 % in male BRCA1-mutation carriers and 5–10 % in male BRCA2-mutation carriers versus 0.1 % in the general population [10]. Mutations in these genes are found in MBC patients with and without a family history of breast and/or ovarian cancer [11, 12]. Therefore, regardless of family history, all men with breast cancer should be routinely screened for BRCA1 and BRCA2 mutations.

Here we report the results of a single-center retrospective analysis of the clinical features, BRCA status, treatments and long-term prognosis of patients affected by MBC.

## Methods

This is a retrospective analysis of all male patients presenting with MBC at the Department of Oncology at University Federico II of Naples between January 1989 and January 2014. We recorded the following data: baseline characteristics (age, height, weight, body mass index, risk factors, family history), tumor characteristics (side affected, stage, histotype, hormonal and HER2 status, and Ki-67 expression), treatment (type of surgery, chemotherapy, endocrine therapy, and/or radiotherapy), BRCA1/2 mutation status (if available), other tumors, and long-term survival. Clinico-pathological data were obtained from medical and pathology reports without performing additional tests. BRCA1 and BRCA2 mutation analysis was performed in 17 MBC patients within the framework of a genetic counseling programs ongoing at our center [13]. BRCA1/2 mutations were classified according to their potential functional effect as recorded in the Breast Cancer Information Core (BIC) database [14] and in the Leiden Open Variation Database (LOVD-IARC) [15].

Data regarding estrogen receptor (ER), progesterone receptor (PgR), Ki-67, and HER2 status of breast tumors were extracted from medical, pathology, or tumor registry records or obtained from the results of immunohistochemical analysis of sections of formalin-fixed, paraffin-embedded primary mammary tumor blocks. HER2 status was assessed by fluorescent in situ hybridization analysis in ambiguous cases (immunohistochemistry score = 2+). According to international guidelines [16], ER and PgR were considered positive if ≥ 1 % of tumor cell nuclei were immunoreactive; whereas Ki-67 was considered high at ≥ 20 % cut-off.

Follow-up and additional diagnostic exams were performed according to clinical practice as for females. Patients were followed with clinical visits, every 6 months up to 5 years and thereafter annually. Patients were contacted by phone to update follow-up and survival status.

All the patients included in this retrospective analysis provided their informed consent in the framework of cancer genetic counseling program regulated and approved by the Local Ethic committee (University ‘Federico II’ of Naples, Prot. 80/00 and 63/02).

### Statistical analysis

Continuous variables are expressed as median with interquartile range (IQR). Categorial variables are expressed as numbers and percentages. Overall survival was calculated with the Kaplan Meier method. A survival analysis for patients who underwent BRCA testing was performed and statistical significance was considered for p value <0.05 calculated with the log rank test. All statistical analyses were performed with SPSS, version 20.0 (SPSS Inc., Chicago, IL, USA).

## Results

**Fig. 1:**
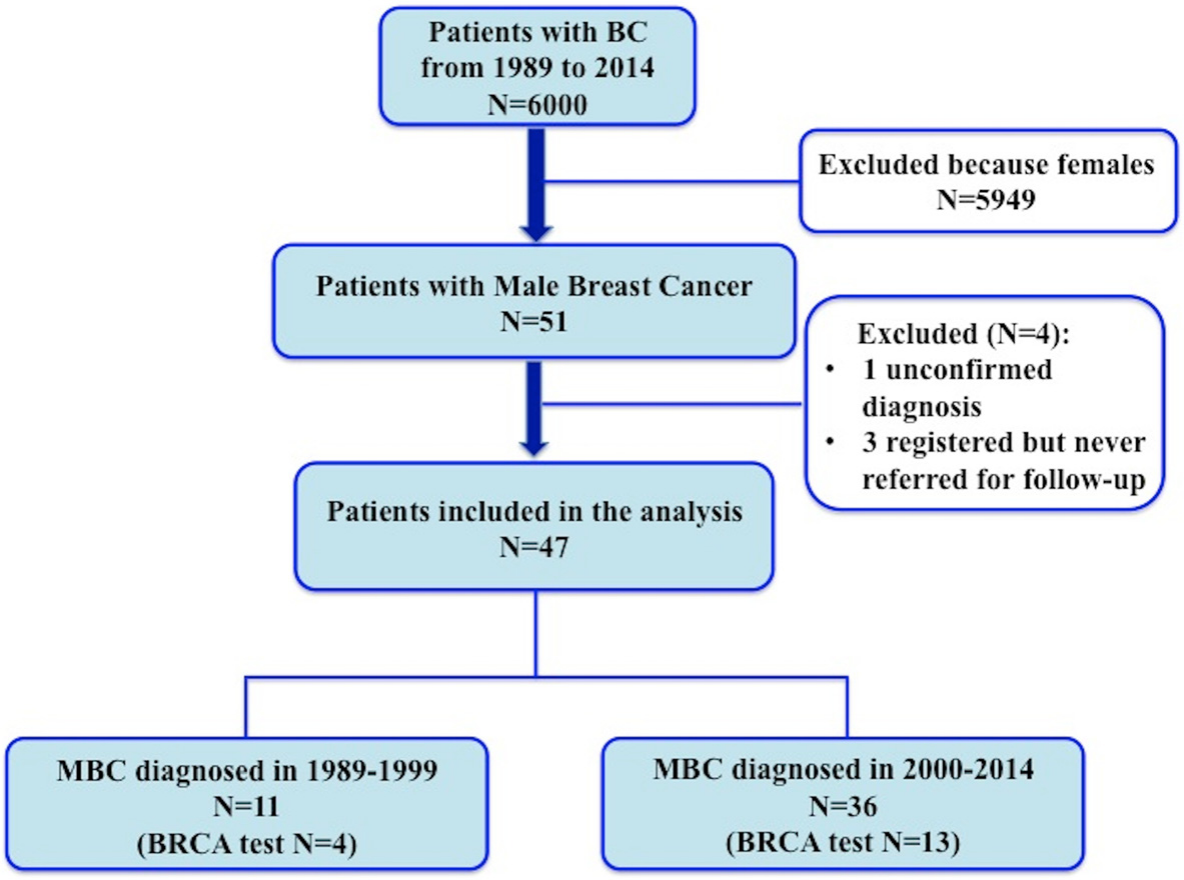
Flow chart of the study. Patients with male breast cancer included in the restrospective analysis

From January 1989 to 1 January 2014, 47 patients received diagnosis and treatment for MBC and were followed at our center (Fig. 1).

### Clinical features

**Fig. 2:**
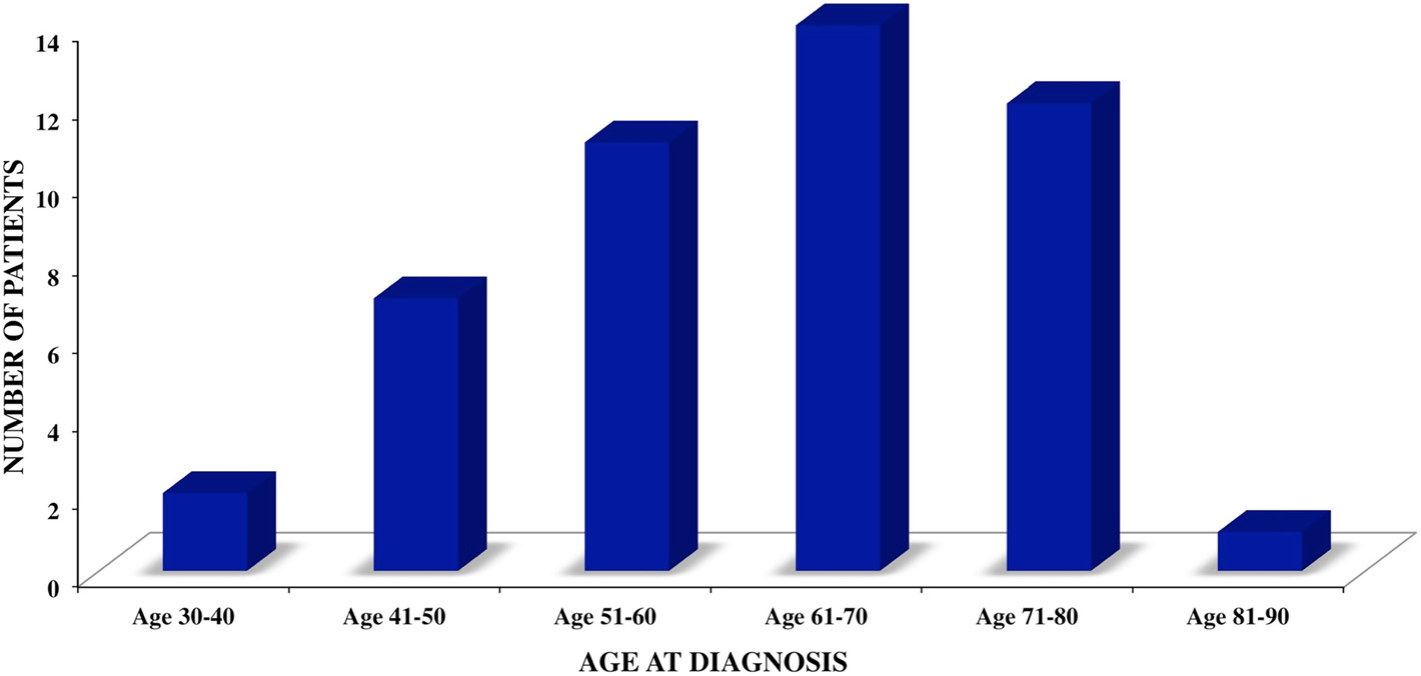
Age distribution in patients with MBC. Distribution of the 47 patients with male breast cancer according to age decades

The baseline and clinical characteristics of the 47 patients and BRCA mutational status (if available) are reported in Table 1. Age at MBC diagnosis ranged between 33 and 82 years (median: 62.0 years [55.0–72.0]). Most patients were over the age of 50 years (80.9 %) and most were diagnosed in the sixth decade of life (Fig. 2). The median value of the body mass index (BMI) was 26.2 [24.2–28.9] kg/m^2^. Apart from 10 patients (21.3 %) classified obese (BMI ≥30 kg/m^2^), no patient had a history of clinical disorders associated with an imbalanced estrogen/androgen ratio such as Klinefelter’s syndrome, liver diseases or testicular abnormalities. Fourteen of the 47 patients (29.7 %) had a family history of breast or ovarian cancer in first-degree relatives. Nine patients (19.1 %) had a second tumor unrelated to MBC (one or two malignancies besides MBC), melanoma and prostate cancer being the most frequent (3 and 2 cases, respectively). In addition, gastric cancer, contralateral breast cancer, thyroid cancer and kidney cancer each occurred in 1 patient.

**Table 1:** Baseline and clinical characteristics of the study population

Characteristics	Patients (*n* = 47)
Age at diagnosis	62.0 [55.0–72.0]
Age at diagnosis	
> 50 years	38 (80.9 %)
≤ 50 years	9 (19.1 %)
Body mass index	26.2 [24.2–28.9]
Family history	14 (29.7 %)
Smoker	10 (21.3 %)
Alcohol intake	10 (21.3 %)
Obesity	10 (21.3 %)
Side affected	
Left	29 (61.7 %)
Right	17 (36.2 %)
Both	1 (2.1 %)
Tumor stage	
I	16 (34.0 %)
II	21 (44.7 %)
III	6 (12.8 %)
IV	1 (2.1 %)
Unknown	3 (6.4 %)
Histotype	
Ductal	45 (95.8 %)
Papillary	1 (2.1 %)
Mixed	1 (2.1 %)
Hormone receptor positive	
ER	38/43 (88.4 %)
PgR	35/43 (81.4 %)
Both	34/43 (79.1 %)
Unknown	4/47 (8.5 %)
ER+/PgR-	4/43 (9.3 %)
ER-/PgR+	1/43 (2.3 %)
Hormone receptor negative	4/43 (9.3 %)
HER-2 positive	11/41 (26.8 %)
Unknown	6/47 (12.8 %)
Triple negative	3/43 (7.0 %)
Nodes status	
Positive	17/44 (38.6 %)
Negative	27/44 (61.4 %)
Unknown	3/47 (6.4 %)
Ki-67 high-level (cut off 20 %)	22/34 (64.7 %)
Unknown	13/47 (27.7 %)
Surgery	
Mastectomy	40 (85.1 %)
Quadrantectomy	7 (14.9 %)
Radiotherapy	14 /43 (32.6 %) (4 unknown)
Chemotherapy	29/41 (70.7 %) (6 unknown)
Endocrine therapy	37/41 (90.2 %) (6 unknown)
Trastuzumab	7/42 (16.7 %) (5 unknown)
Relapse	9/40 (22.5 %) (6 unknown)
Other tumors	9 (19.1 %)
BRCA test	
No	30 (63.8 %)
Yes	17 (36.2 %)
BRCA mutation	
BRCA 1	1 (5.9 %)
BRCA 2	5 (29.4 %)
Unknown variant sequence	1 (5.9 %)
Negative	10 (58.8 %)
Death	7 (14.9 %)
No BRCA test	4 (57.1 %)
BRCA 1	1 (14.3 %)
BRCA 2	2 (28.6 %)
Follow-up	Median 2660 [1065–4452] days (7.3 years)

Continuous variables are reported as median and interquartile range

Abbreviations: *ER* estrogen receptor, *PgR* progesterone receptor

### Clinico-pathological features

Most tumors were at early TNM stage: stage II (44.7 %) and 16 were stage I (34.0 %). Only one case was bilateral (2.1 %).

Pathologic confirmation was available for all 47 patients. Infiltrating ductal carcinoma was the most common histologic subtype (45 patients; 95.8 %), whereas papillary carcinoma was diagnosed in 1 patient and mixed (tubular-ductal) carcinoma was diagnosed in the remaining patient. Hormonal status was available for 43 patients: 38 (88.4 %) were ER+ and 35 (81.4 %) were PgR+; the distribution of both hormone receptors was as follow: 34 cases were ER+/PgR+ (79.1 %), 4 were ER+/PgR- (9.3 %), 1 was ER-/PgR+ (2.3 %) and 4 were ER-/PgR- (9.3 %). HER2 status was known in 41 patients and was positive in 11 cases (26.8 %). Triple negative phenotype was revealed in 3 cases (7 %). In 22 patients (64.7 %) the tumor had high proliferative activity, namely, Ki67 ≥ 20 %.

### Treatments

Data on the surgical approach were available for all patients. The most frequently used surgical approach was mastectomy (40 patients, 85.1 %), whereas the remaining 7 patients (14.9 %) underwent quadrantectomy. Positive axillary nodes were found in 17 of the 44 patients (38.6 %) who underwent axillary dissection (defined as I/II lymph nodes level removal). Fourteen of 43 patients (32.6 %) received post-operative local radiotherapy, while data were not available for 4/47 patients. The dose used for adjuvant irradiation was 50 Grey (Gy) in 25–28 fractions (2 Gy/fraction) with an additional boost of 10 Gy if clinically indicated. The radiotherapy was performed using the linear accelerator with 6 megavolts photons.

Among patient for whom data are available, systemic medical treatment consisting of adjuvant chemotherapy was administered to 29/41 patients (70.7 %): 17 (58.6 %) received antracycline-based treatment (5 patients received both antracycline and taxane); 7 (24.1 %) received CMF (cyclophosphamide, methotrexate, 5-fluoracil) combination chemotherapy and 5 (17.2 %) received only taxane. Adjuvant endocrine therapy was administered in 37/41 patients (90.2 %): tamoxifene alone in 29/37 patients (78.4 %), and 6 patients (16.2 %) were treated with aromatase inhibitors (AIs). The regimen based on tamoxifene followed by AI was used in 2 cases (5.4 %). Ten of 41 patients (24.4 %) received only endocrine therapy as adjuvant; 27 patients (65.9 %) received both chemotherapy and endocrine therapy in a sequential manner. Moreover, 7 of 42 patients (16.7 %) received trastuzumab therapy, in 1 case in a metastatic setting.

All patients completed adjuvant treatment program and no relevant side effects, treatment intolerance or patients’ refusal were recorded.

### Follow-up

Median follow up was 89 months (2660 days, 7.3 years) (Table 1). During follow-up, 9 patients of 40 (22.5 %), for whom data about follow-up was available, experienced relapse at the following sites: thoracic wall (*n* = 4), bone (*n* = 4), lung (*n* = 4), breast (*n* = 1), brain (*n* = 1), axillary nodes (*n* = 1) and adrenal gland (*n* = 1). The BRCA test was performed in 17 patients (36.2 %). Ten patients (58.8 %) were wild-type for BRCA genes, 1 was a carrier of BRCA1 (5.9 %) and 5 (29.4 %) of BRCA2 mutations, while 1 patient (5.9 %) had an unknown variant sequence. The characteristics of these 17 patients are reported in Table 2. The BRCA1 mutation-positive MBC patient was <40 years old at diagnosis and had a first-degree family history of breast and ovarian cancer. No other risk factors were present. He had an invasive ductal carcinoma, stage II, lymph node positive, HER2– and ER+/PgR+, and low Ki-67 levels. The disease relapsed after adjuvant treatment (chemotherapy and endocrine therapy) and the patient developed a second tumor (thyroid cancer). This patient died at 59.8 months of follow-up. The median age at diagnosis of the 5 BRCA2-positive patients was 72.0 [62.0–72.5] years, and 2 had a positive first-degree family history of breast and/or ovarian cancer (Table 2). No other risk factors were present. MBC was an invasive ductal carcinoma in all cases, stage I (3 cases) and III (2 cases); all cases were ER+/PgR+, with high levels of Ki67, and only 1 was HER2+. Nodal involvement was observed in 2 patients. One patient relapsed after adjuvant treatment (chemotherapy and endocrine therapy) and 2 developed a second tumor (one case of prostate cancer, and one of prostate and kidney cancer) (Table 3).

**Table 2:** Characteristics of patients with known BRCA status

Characteristics	BRCA-negative	BRCA1-positive	BRCA2-positive					BRCA UVS
	*n* = 10	*n* = 1	Patient 1	Patient 2	Patient 3	Patient 4	Patient 5	*n* = 1
Age at diagnosis	61.0 [53.8–67.3]	30–40	60–70	70–80	70–80	60–70	70–80	70–80
BMI	26.2 [23.6–32.0]	22.8	24.5	33.6	27.0	23.2	31.4	25.8
Family history	50 %	Yes	No	Yes	No	No	Yes	No
Smoke	0 %	No	Yes	No	Yes	No	No	No
Alcohol intake	10 %	No	Yes	No	No	No	Yes	No
Obesity	20 %	No	No	Yes	No	No	Yes	No
Side	Left (70 %); Right (30 %)	Right	Right	Right	Left	Both	Left	Left
Stage	I (20 %); II (70 %); III (10 %)	II	I	III	III	Unknown	I	I
Histotype	Ductal (100 %)	Ductal	Ductal	Ductal	Ductal	Ductal	Ductal	Ductal
HR status	ER+/PgR+ 9	ER+/PgR	ER+/PgR	ER+/PgR	ER+/PgR	ER+/PgR	ER+/PgR	ER+/PgR
	Unknown 1	+	+	+	+	+	+	+
Node status, positive	50 %	Yes	No	Yes	Yes	No	No	No
HER-2 positive	10 % Unknown (10 %)	No	No	No	Yes	No	No	No
Ki-67 high-level (cutoff 20 %)	60 % Unknown (10 %)	No	Unknown	Yes	Yes	Yes	Yes	Yes
Surgery	M 90 %	M	M	M	Q	M	M	M
Radiotherapy	50 %	No	No	No	Yes	No	No	No
Chemotherapy	60 % Unknown (10 %)	Yes	Yes	Yes	Unknown	Yes	No	Yes
Endocrine therapy	90 % Unknown (10 %)	Yes	Yes	Yes	Unknown	Yes	Unknown	Yes
Trastuzumab	10 % Unknown (10 %)	No	No	No	Unknown	No	No	No
Relapse	20 %	Yes	No	Yes	Yes	Unknown	No	No
Other tumors	20 %	Yes	No	No	Yes	Yes	No	No
Follow-up (days)	3152 [701–6735]	1795	3845	2892	1096	7069	1031	918

For single patients, age is reported as decade range to maintain participant confidentiality

Abbreviations: *BMI* body mass index, *ER* estrogen receptor, *HR* hormone receptor, *M* mastectomy, *PgR* progesterone receptor, *Q* quadrantectomy, *UVS* unknown variant sequence

**Table 3:** Characteristics of patients who died

Characteristics	Patient 1	Patient 2	Patient 3	Patient 4	Patient 5	Patient 6	Patient 7
Age at diagnosis	40–50	50–60	30–40	70–80	70–80	70–80	50–60
Body mass index	20.7	27.0	22.8	27.0	31.4	29.0	25.0
Family history	No	No	Yes	No	Yes	No	No
Smoke	No	No	No	Yes	No	No	No
Alcohol intake	No	Yes	No	No	Yes	No	No
Obesity	No	No	No	No	Yes	No	No
Side	Left	Left	Right	Left	Left	Right	Left
Stage	IV	II	II	III	I	III	Unknown
Histotype	Ductal	Ductal	Ductal	Ductal	Ductal	Ductal	Ductal
HR status	ER+	ER-/PgR-	ER+/PgR+	ER+/PgR+	ER+/PgR+	ER+/PgR+	Unknown
HER-2 positive	Yes	Unknown	No	Yes	No	No	Unknown
Ki-67 high-level (cutoff 20 %)	Unknown	Unknown	No	Yes	Yes	Yes	Unknown
Surgery	M	M	M	Q	M	M	M
Radiotherapy	Yes	No	No	Yes	No	No	No
Chemotherapy	Yes	Yes	Yes	Unknown	No	Yes	No
Endocrine therapy	Yes	No	Yes	Unknown	Unknown	Yes	No
Trastuzumab	Yes	No	No	Unknown	No	No	No
Relapse	Yes	No	Yes	Yes	No	Unknown	Yes
Other tumors	Yes	No	Yes	Yes	No	No	Yes
BRCA mutation	No test	No test	BRCA 1	BRCA 2	BRCA 2	No test	No test
Follow-up (days)	2465	5706	1795	1096	1031	173	3713

For single patients, age is reported as decade range to maintain participant confidentiality

Abbreviations: *ER* estrogen receptor, *HR* hormone receptor, *M* mastectomy, *PgR* progesterone receptor, *Q* quadrantectomy

**Fig. 3:**
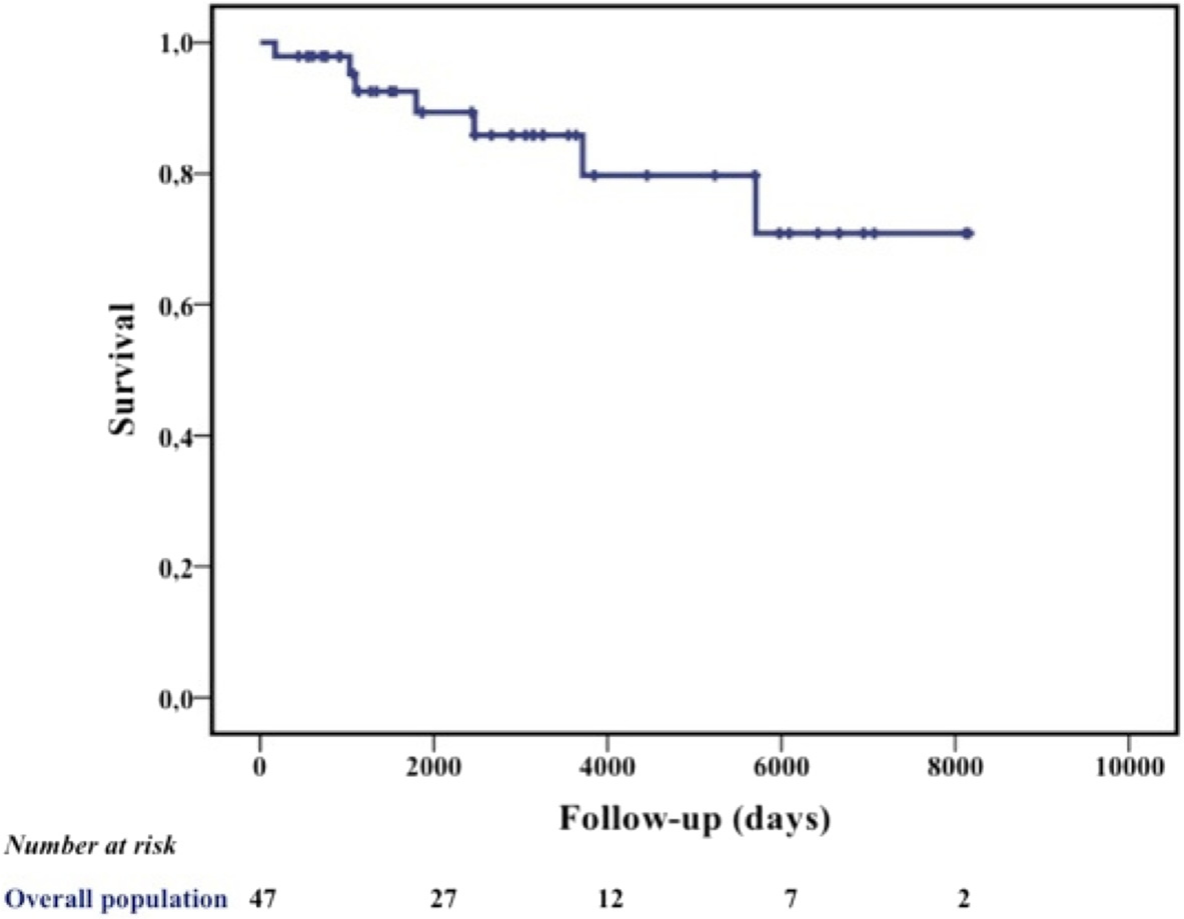
Survival in patients with MBC. Kaplan-Meier survival estimates in the 47 patients with MBC included in the study

Seven of 47 patients (15 %) died during follow-up, and the estimated long-term survival was about 90 % at 5 years, 80 % at 10 years and 70 % at 18–20 years (Fig. 3, Table 3).

**Fig. 4:**
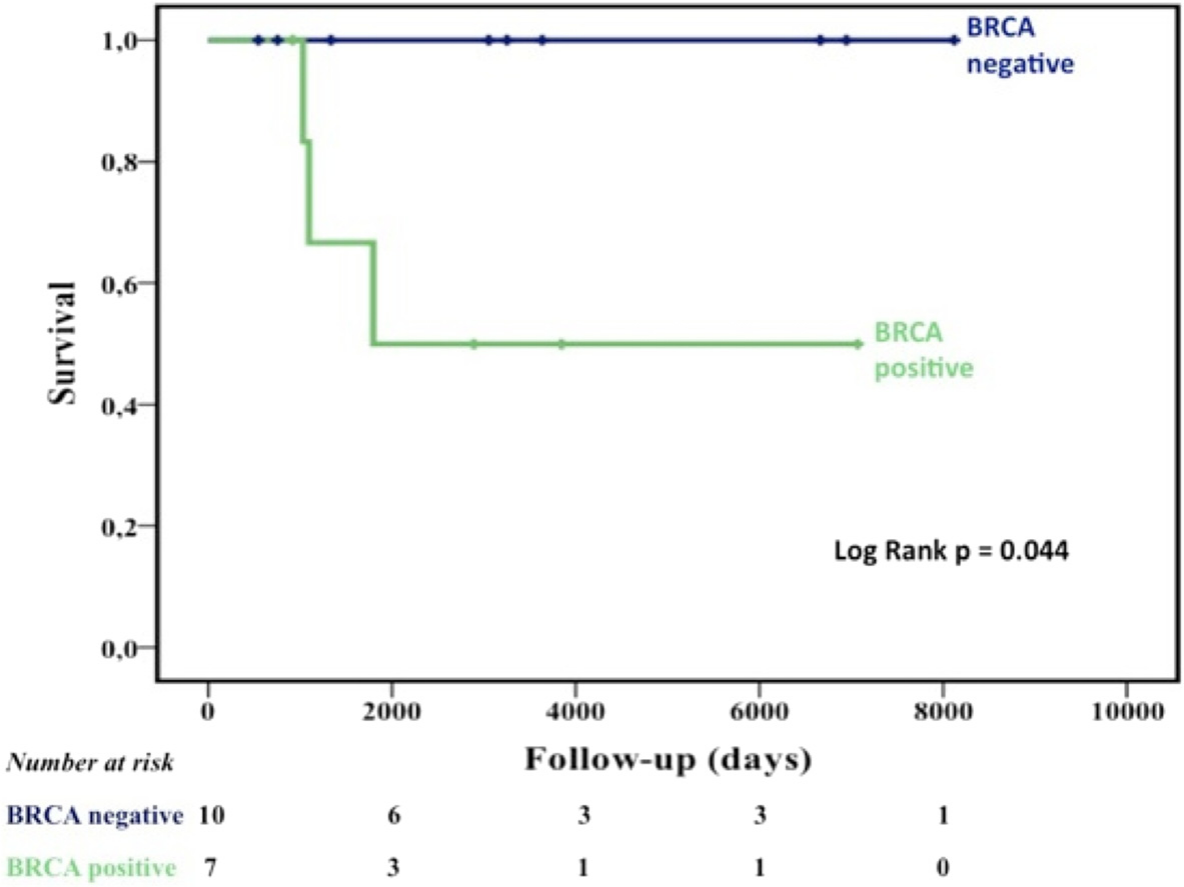
Survival in patients with known BRCA mutation status. Kaplan-Meier survival estimates in the 17 patients underwent to BRCA test and stratified for mutational status

Among the patients with a known BRCA status, 3 patients died and all were mutation carriers. Survival was significantly lower in BRCA-mutated patients (*p* = 0.04; Fig. 4).

## Discussion

In this single-center retrospective study, we report the characteristics of 47 patients with MBC and analyze the patients’ clinical-pathological features, associated risk factors, oncological and surgical treatments and long-term survival. We also evaluated survival in 17 patients who underwent genetic testing for BRCA1 and BRCA2 mutations.

Median age of our patients at diagnosis was 62 years, which is similar to previously reported data [17]. Regarding the risk factors associated with MBC, 10/47 of our patients (21.3 %) had a history of obesity. Obesity is known to be a risk factor for MBC, and obese men have an increased risk of about 30 % of breast cancer, similar to that of postmenopausal women [18]. In men, obesity is associated with high estrogen levels, and low levels of testosterone and sex hormone binding globulin, thereby leading to greater estrogen bioavailability [19, 20].

No patients in our series was affected by Klinefelter’ syndrome, hormonal alterations or chronic liver disease, which are known risk conditions for MBC [4].

Among genetic risk factors, we found that a family history of breast or ovarian cancer is as relevant in men as in women. A positive family history of breast or ovarian cancer was recorded in 29.7 % of our patients, which is slightly higher percent than reported previously [3]. Mutational analysis for the BRCA1/2 genes was performed in 17/47 patients (32.2 %). BRCA2 mutations were found in five cases (29.4 %) and BRCA1 mutations in only one patient. Despite the low percentage of patients who underwent genetic testing, the greater association with BRCA2 mutations compared to BRCA1 mutations in our MBC patients is in line with a previous report [5].

Carriers of BRCA1 and BRCA2 mutations genes are at an increased risk for cancer at body sites other than breast, such as prostate, stomach, pancreatic cancers and melanoma [3]. According to previous data, about 19 % of our patients developed a second tumor [3].

The role of family history, germinal mutation of BRCA1/2 genes, risk of cancers associated to breast and ovarian cancer syndrome related to BRCA1/2 genes, suggest that cancer genetic counseling for MBC patients should contribute to better clarify genotype-phenotype correlations and to personalize surveillance strategies according to international guidelines (http://www.nccn.org/professionals/physician_gls/f_guidelines_nojava.asp#site).

We found that T stage at diagnosis was heterogeneous, but 44.7 % of our patients were at stage II with axillary lymph node involvement in almost half of these cases (40.5 %). Despite the small sample size, stage at diagnosis was in line with previously described in a larger report [21]. Because of the lower level of awareness among men, and a low index of suspicion for the disease there is often a diagnostic delay. Moreover, a screening program is not available for men because of the low lifetime risk [9].

In the present study, there was a left-sided preponderance over the right side (61.7 % versus 36.2 % respectively) consistent with a previous report [21]. As expected, the most prevalent histological subtype was invasive ductal carcinoma. Our data confirm the high rate of hormone-receptor positivity associated with MBC [22]. In fact, 88.4 % of our MBC patients were ER+ and 81.4 % PgR+. We found that 26.8 % of our patients were HER2 positive. Data on HER2 status in MBC is very heterogeneous, with HER2 overexpression rates ranging from 2 % to 42 % [23–26].

The most frequently used surgical procedure for loco-regional treatment of MBC is modified radical mastectomy (MRM) and it was performed in 40 of our patients (80.5 %), which is consistent with a previous study [27]. This approach is probably preferred given the anatomic characteristics of male breast tissue, the limited surgical sequelae and the better cosmetic outcome compared to other procedures [3, 28]. Most of our patients underwent axillary dissection, as suggested in the literature [5]. In recent years, the technique of sentinel lymph node (SLN) is a reliable tool in MBC patients, as shown in recent experiences [29–31].

The criteria for administration of post-surgical radiation are usually extrapolated from data obtained in women, due to the absence of controlled trials [32]. In the present series, postoperative radiotherapy was performed in 14 of 43 patients (32.6 %) and 7 of them received post-mastectomy radiotherapy. Several studies showed that radiation reduces the post-operative loco-regional recurrence rate [33–36], but only one study demonstrated a survival benefit [36].

In our study, tamoxifen was the most frequent adjuvant hormone therapy and AIs alone were used in 6 (16.2 %) patients. Endocrine therapy was found to be beneficial in small retrospective studies [37–39]. In the study by Goss et al. [39] adjuvant hormone treatment with tamoxifen significantly improved disease-free and overall survival, thereby representing the standard of care. The role of AIs in male patients is not as clear as it is for women. Monotherapy with AIs does not completely restrain estrogen production because they do not inhibit the testicular production of estrogen, which represents 20 % of circulating estrogen [40]. Moreover, AI administration causes an increase in the levels of luteinizing hormone and follicle stimulating hormone that could lead to an increase in the substrate for aromatization, suggesting that AIs could be used in combination with a luteinizing hormone-releasing hormone analog [41]. Aromatase inhibitors are mainly used in metastatic patients who are resistant to tamoxifen or with contraindications to tamoxifen therapy [41].

The role of chemotherapy in MBC is not well defined and only CMF has been prospectively evaluated in the adjuvant setting [42, 43]. In the study conducted by Giordano et al., 63 % of patients undergoing systemic treatment received chemotherapy (alone or in combination with hormone therapy) and anthracycline-based chemotherapy, more frequently used than CMF (81 % versus 16 %), had a reduced risk of death [44]. In our series, adjuvant chemotherapy was administered to 29 patients (70.7 %) and antracycline-based treatment (with or without taxane) was the preferred regimen. The patient’s evaluation was performed considering different clinical and pathological features such as tumor stage, ER/PgR and Ki-67 level, HER-2 status, age and comorbidities, as commonly done in female BC patients, in order to select those who may benefit from systemic treatment. The high percentage of patients undergoing chemotherapy could be explained by the following considerations: 1) 40 % of patients had nodal positive disease; 2) patients with hormone-receptors negative disease were considered at high-risk and received chemotherapy, particularly, those patients with triple negative phenotype (7 % of patients); 3) patients with HER-2 positive disease were treated with trastuzumab plus chemotherapy (roughly 27 % of patients); 4) 65 % of patients were found to have high levels of Ki-67; 5) no patients refused treatment or presented age-related comorbidities contraindicating chemotherapy; 6) the majority of the patients included in our series received diagnosis of MBC after 2000s (Fig. 1).

In our series, the estimated long-term survival was about 90 % at 5 years, 80 % at 10 years and 70 % at 18–20 years. Percentage of long-term survival is higher for our patients compared to data reported in literature. MBC has a worse overall prognosis than female breast cancer, namely, an overall 5-year survival rate of 40–65 % versus 85 % in women [45]. However, when matched for stage, age and prognostic factors, the prognosis is similar [45]. The worse outcome in men seems primarily due to the presence of more advanced disease at diagnosis, and gender itself does not seem to be a prognostic factor [46]. Evidence on survival in MBC is quite small compared with female BC and the wide range of survival described likely reflects the heterogeneity of disease stage and different treatments strategies across time. Our high rates of survival might be related to several aspects such as patient characteristics, tumor features, and treatments strategies adopted. To better put our results into the context of previous literature, the following considerations should be taken into account: 1) the most important prognostic factors in MBC seem to be patient age, tumor stage and lymph node status. Fentiman et al. reported 5-year survival rates of 75–100 % for stage I, 50–80 % for stage II, and 30–60 % for stage III [47]. Additionally, some data showed that 40 % of patients with MBC will die for other causes [3], likely reflecting the influence of comorbidities and older mean age at diagnosis. Most of our patients were diagnosed in the sixth decade of life and at early TNM stage (approximately 79 % stage I-II), without significant age-related comorbidities contraindicating the treatment; 2) 90 % of our patients received hormone therapy, mainly tamoxifen which demonstrated to decrease recurrence and improve overall survival [39]; 3) 70 % of our patients received adjuvant chemotherapy and previous studies showed that patients undergoing chemotherapy have survival benefits compared to those without chemotherapy [45, 46]. Moreover, most of our patients received antracycline and/or taxanes rather than CMF, and this was previously found to be associated with better survival in female disease; 4) trastuzumab was used in all HER2 positive disease and it is well-known to improve survival in female BC.

Obviously, we cannot draw definitive conclusions on the impact of each factor on the overall survival, however, this experience could be useful for the current limited knowledge on MBC and for driving future prospective studies.

In women, BRCA-associated BC tends to manifest specific genotype–phenotype correlations [48], whereas little is known about the phenotype characteristics of BRCA-associated MBC. A recent study identified more high-grade, progesterone-receptor negative, HER2-positive disease in male patients who carried BRCA2 mutations [9], and earlier research found poorer prognosis in men with BRCA2-associated tumors [9]. Interestingly, we found that patients with BRCA mutation showed a low survival compared with BRCA wild-type. Despite small size, this is the second study on association with prognosis and BRCA status. Our data are consistent with the poorer prognosis previously reported [9].

One of the limits of this study is its retrospective nature and the small number of patients enrolled. However, it is difficult to conduct randomized trials or large multicenter studies because of the rarity of MBC. Other limitations are: 1) the presence of some missing data; and 2) the availability of BRCA status just for 17 patients. However, many of our patients have a long-term follow-up and some were referred to our center in the 1990s when BRCA testing was not a routine procedure.

## Conclusion

This study has shown a high long-term survival rate in patients with MBC compared with other studies. A significantly reduced survival rate was registered in the subgroup of patients carrying BRCA1/2 mutations in line with the poorer prognosis previously reported in the same setting. MBC remains a challenge and future larger studies are warranted. However, given the low incidence of the disease, prospective studies are difficult to plan, and retrospective data collections such as our series play an important role in acquiring information about MBC.

## Abbreviations

AIs, aromatase inhibitors; BIC, breast cancer information core database; BMI, body mass index; CMF, cyclophosphamide, methotrexate, 5-fluoracil; ER, estrogen receptor; HER2, human epidermal growth factor receptor 2; IQR, interquartile range; Ki-67, proliferation index; LOVD-IARC, Leiden Open Variation Database; MBC, male breast cancer; MRM, radical mastectomy; PgR, progesterone receptor; SLN, sentinel lymph node
